# Supplementary material for: ExplorePipolin: reconstruction and annotation of piPolB-encoding bacterial mobile elements from draft genomes
Source: Bioinform Adv. 2022 Aug 10;2(1):vbac056. doi: 10.1093/bioadv/vbac056 (PMC9710591; doi:10.1093/bioadv/vbac056)
Supplement: vbac056_Supplementary_Data [file vbac056_supplementary_data.pdf]

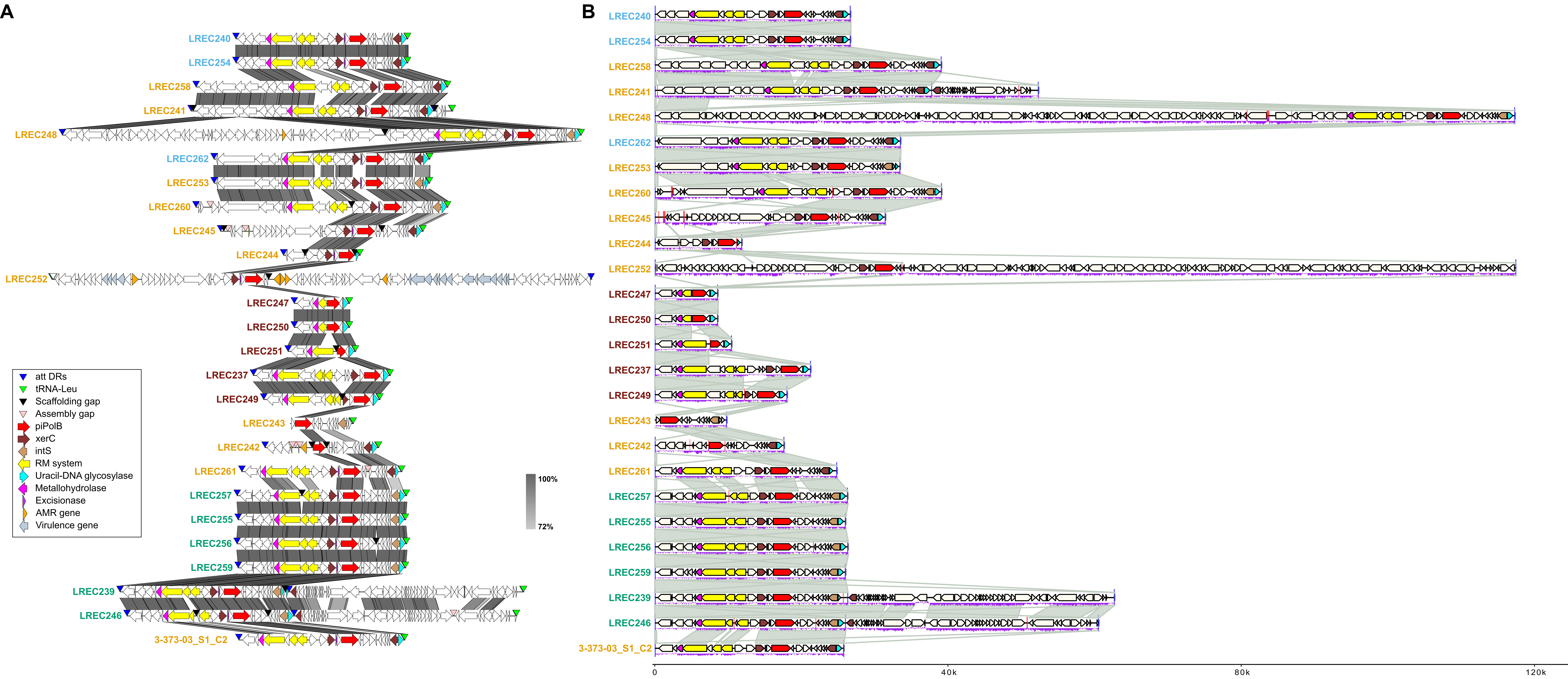

Supplementary Figure 1.

Comparison between E. coli pipolin structures from LREC collection (Flament-Simon et al. 2020) as originally delineated (A) and with the current version of ExplorePipolin (B). Pipolins in panel B were plotted using gggenomes (Hackl,T. and Ankenbrand,M.).
